# 

**Published:** 1901-03

**Authors:** 


### PERSONALS.

Dr. Leonard Pearson, State Veterinarian of Pennsylvania, addressed the Guernsey Cattle Breeders' Association of the Keystone State at Philadelphia.

The Collins Veterinary Medical College of Nashville, Tennessee, is not meeting with the success which its "promoter" hoped for, as there are only two students in attendance this session as against three last session.

Dr. Alex. Glass, of Philadelphia, has again been selected as the official photographer of the Corinthian Yacht Club. His love of aquatic pastime is unabated, but he yearns for the summer yachting season.

Dr. R. S. Huidekoper was a visitor to Philadelphia early in February.

After an absence of almost a year Dr. Joseph Plaskett has resumed the practice of his chosen profession in Nashville, Tennesse. During the past year Dr. Plaskett has landed three cargoes of live-stock from this country to the British government in South Africa.

Dr. E. W. Hammond, of the class of '99, Veterinary Department, McGill University, has recently been appointed bacteriologist to the City Dairy Company, of Toronto, Canada.

Dr. James T. Stuart, of Cleveland, Ohio, has removed to Glenville, in the same State, where he will continue the practice of veterinary medicine.

Dr. J. P. Foster, of Selby, South Dakota, has just receivd the appointment of State veterinarian for his State.

Dr. Porte Crego has been elected secretary of the Aurora (Illinois) Driving Park Association. Dr. Crego takes an active interest in the fast roadsters.

Dr. F. C. Grenside, of New York, is mentioned among others as a judge in the saddle classes at the Boston Horseshow.

State Veterinarian Pearson, of Pennsylvania, made a flying trip to Northampton County, Pennsylvania, in February, to make an investigation of a fatal cattle disease.

Dr. B. M. Underhill, of Media, Pa., is now sojourning for a time at Knoxville, Iowa.

Dr. Cecil French, of Washington, D. C., will visit Europe the coming summer, visiting the leading veterinary schools and especially their canine departments.

Dr. H. D. Gill takes an active interest in the Road Drivers' Association of New York City. At a recent meeting the initiation fee and dues were raised to $5 each and a place made for non-resident members.

Dr. H. Thomson, formerly of Shabbona, Illinois, is now located at Newman Grove, Nebraska.

Dr. Owen Reist, of Kossuth, Ontario, has been returned by acclamation to the position of township councillor.

Dr. W. H. Wheeler, of Stamford, N. Y., takes an active interest in horses of speed and enjoys a holiday each year after them on the tracks.

Dr. James S. Cattanach and son, of New York City, take an active interest in the Rider and Driver's Mounted Police Medal and Hospital Fund.

Dr. J. Stewart Lacock, of Alleghany, Pa., officiated as veterinarian of the Duquesne Kennel Club at Pittsburg in March.

Dr. Tait Butler, recently of Milwaukee, has succeeded Dr. Paul Fischer at Manhattan, Kansas, as Professor of Veterinary Science and Biology and State Veterinarian.

Dr. F. F. Hoffman, of Brookville, Pa., has been seriously ill, but is now on the way to recovery.

Dr. Wm. Henbeit Lowe, of Paterson, N. J., takes an active interest in the religious affairs of his city and is a vestryman of an Episcopal church of that city.

Dr. Winchester's tribute to the late Dr. Clement—" Thus passes a light from us whose work can well be used for a guide for many."

Dr. W. Horace Hoskins was a visitor to the national capital in February, spending some hours with Dr. Hurlbert Young, of the " City of Magnificent Distances."

Dr. E. K. Shaub, of Lancaster, Pa., is taking a course at the Medico-Chirurgical College at Philadelphia.

